# Thirst Perception and Osmoregulation of Vasopressin Secretion Are Altered During Recovery From Septic Shock

**DOI:** 10.1371/journal.pone.0080190

**Published:** 2013-11-06

**Authors:** Shidasp Siami, Andrea Polito, Raphael Porcher, Tarik Hissem, Anne Blanchard, Catherine Boucly, Robert Carlier, Djillali Annane, Jean-Philippe Haymann, Tarek Sharshar

**Affiliations:** 1 Department of Intensive Care Medicine, Sud Essonne Hospital, Etampes, France; 2 General Intensive Care Medicine, Assistance Publique Hôpitaux de Paris, Raymond Poincaré Hospital, University of Versailles Saint-Quentin en Yvelines, Garches, France; 3 Biostatistics and Medical Computer Science Department, Saint-Louis Teaching Hospital, Paris, France; 4 Clinical Investigation Centre, Assistance Publique Hôpitaux de Paris, European Georges Pompidou Teaching Hospital, University René Descartes and INSERM CIC9201, Paris, France; 5 Department of Biochemistry, Assistance Publique Hôpitaux de Paris, Raymond Poincaré Hospital, University of Versailles Saint-Quentin en Yvelines, Garches, France; 6 Department of Radiology, Assistance Publique Hôpitaux de Paris, Raymond Poincaré Hospital, University of Versailles Saint-Quentin en Yvelines, Garches, France; 7 Department of Functional Investigations, Assistance Publique Hôpitaux de Paris, Tenon Teaching Hospital, University of Pierre et Marie Curie-Paris 6 and INSERM U702, Paris, France; St. Joseph's Hospital and Medical Center, United States of America

## Abstract

**Objective:**

Vasopressin (AVP) secretion during an osmotic challenge is frequently altered in the immediate post-acute phase of septic shock. We sought to determine if this response is still altered in patients recovering from septic shock.

**Design:**

Prospective interventional study

**Setting:**

Intensive care unit (ICU) at Raymond Poincaré and Etampes Hospitals.

**Patients:**

Normonatremic patients at least 5 days post discontinuation of catecholamines given for a septic shock.

**Intervention:**

Osmotic challenge involved infusing 500 mL of hypertonic saline solution (with cumulative amount of sodium not exceeding 24 g) over 120 minutes.

**Measurements and main results:**

Plasma AVP levels were measured 15 minutes before the infusion and then every 30 minutes for two hours. Non-responders were defined as those with a slope of the relation between AVP and plasma sodium levels less than < 0.5 ng/mEq. Among the 30 included patients, 18 (60%) were non-responders. Blood pressure and plasma sodium and brain natriuretic peptide levels were similar in both responders and non-responders during the course of the test. Critical illness severity, hemodynamic alteration, electrolyte disturbances, treatment and outcome did not differ between the two groups. Responders had more severe gas exchange abnormality. Thirst perception was significantly diminished in non-responders. The osmotic challenge was repeated in 4 non-responders several months after discharge and the abnormal response persisted.

**Conclusion:**

More than half of patients recovering from septic shock have an alteration of osmoregulation characterised by a dramatic decrease in vasopressin secretion and thirst perception during osmotic challenge. The mechanisms of this alteration but also of the relationship between haematosis and normal response remain to be elucidated.

## Introduction

It has been proposed that relative arginine-vasopressin (AVP) deficiency contributes to hypotension in septic shock [1], which is a life threatening complication of infection with a mortality rate ranging from 35 to 70% [2]. AVP regulates circulating volume through renal V2 receptors, smooth muscle tone through arteriolar V1 receptors and the hypothalamo-pituitary adrenal (HPA) axis [3]. Plasma AVP levels have been shown to decrease during septic shock and after 36 hours to be, inappropriately within the normal range despite hypotension in about a third of septic shock patients [4]. One mechanism for this could be an alteration of the osmoregulation of AVP release. 

We designed a project to determine, in two separate cohorts, whether AVP secretion during an osmotic challenge is altered in the post-acute phase (i.e. 3 days from onset) and after recovery from septic shock, respectively. We opted for two separate cohorts as we considered that serial osmotic tests could not easily be achieved because of the high mortality rate between post-acute and recovery phases, the discharge of patients recovering rapidly from septic shock and the occurrence of a contraindication to hyperosmolar loading.

Regarding the first objective, we have previously reported that AVP secretion during an osmotic challenge performed during the post-acute phase (i.e. 3 days from onset) of septic shock is dramatically reduced in half patients. These non-responders were more likely to have bacteraemia or liver dysfunction. They had been more frequently referred from ward or undergone surgery. Responders had a significantly lower PaO_2_/FiO_2_ ratio, indicating a severe impairment of gas exchange [5]. 

We report here the second part of the project, which consisted of assessing change in plasma AVP during an osmotic challenge in patients who had recovered from septic shock. In addition to our previous study, post-hypophyseal hypersignal on magnetic resonance imaging [6] and thirst were assessed prior to and during osmotic challenge, respectively. Evaluating the posterior hypophyseal content of AVP using MRI would allow us to determine if absence of response was secondary to abnormal hypophyseal emptying. If altered, the latter would suggest that osmoreceptors are dysfunctioning, as they are required to stimulate thirst centre [7]. 

We would like to emphasize that the purpose of this project was not to assess whether osmoregulation of vasopressin secretion is impaired in non-septic critically ill patients. As mentioned above, inappropriateness of plasma vasopressin levels has been reported only in septic patients and this was the rationale for testing the osmoregulatory pathway in this specific patient population. 

## Patients and Methods

### Ethical approval

The study protocol (EUDRACT N°051015) was approved by the *Comité de Protection des Personnes de Saint-Germain en Laye* (France). Written informed consent was obtained from patients or their closest relative where necessary.

### Patients

From January 2010 to June 2011, all adults patients who were admitted with septic shock to either of the two participating general intensive care units (ICU) (university-affiliated Raymond Poincaré Hospital, Garches, France; and community hospital, Sud-Essonne, Etampes, France) and who survived at least five days after the discontinuation of vasopressor therapy were included. Criteria for septic shock were those established by the American College of Chest Physicians/Society of Critical Care Medicine criteria [8]. Exclusion criteria were pregnancy, preexisting autonomic dysfucntion or neuroendocrine disease, neurosurgical patients, a contraindication to osmotic challenge (basal plasma sodium level of <130 or >140 mmol/L, a central venous pressure of >18 mm Hg if available, evidence or severe heart failure or infusion of vasopressin). The patients in the present study do not include any participants from our previous work on osmoregulation in the post-acute phase of septic shock [5]. 

### Osmotic Challenge and Measurement of Plasma Vasopressin Levels

The osmotic challenge was adapted from a previously validated test [9] and performed at least 5 days after the discontinuation of vasopressor therapy. Where possible it was also planned to repeat the test after discharge from hospital to see whether any abnormal response persisted. Osmotic challenge consisted on infusion of hypertonic 0.85 M saline solution (5%) at a rate of 0.06 mL per kg of bodyweight per minute over 2 hrs, by either peripheral or central venous catheter. Central venous catheter was never maintained or performed for the purpose of this study (only 6 patients had one at time of test). Overall, the cumulative amount of sodium infused did not exceeded 24 g. Systolic (SAP) and mean arterial (MAP) pressures, heart rate (HR), and arterial oxygen saturation (SaO_2_) were monitored continuously, and Glasgow Coma Scale scores and urine output (mL/hr) were serially recorded. Blood was sampled 15 mins before and 15 mins, 45 mins, 75 mins, and 105 mins after saline infusion. Blood samples were centrifuged and stored at -80°C until analysed. Plasma concentrations of sodium (mmol/L), glucose (mmol/L), urea (mmol/L), and osmolality (mOsml/L) were measured, using conventional tests. Plasma AVP levels were measured by radioimmunoassay [10,11]. 

As in our previous study, nonresponders were defined as those with a slope for the relation between plasma levels of AVP and sodium, of <0.5 ng/mmol [12]. This cut-off was not arbitrary but derived from dose-response curves obtained in both young and elderly healthy subjects [13], which reported that slope of plasma vasopressin increase during an equivalent osmotic challenge was always above > 0.5. 

Plasma levels of Brain Natriuretic Peptide (BNP) were assessed before and after the osmotic challenge using enzyme-linked immunosorbent assays (Abbott Diagnostics, Abbott Park, IL). 

### Assessment of thirst

Thirst was assessed before and at the end of the osmotic challenge in conscious patients by asking them to rate its intensity on a 10 cm visual analogue scale bounded on the left by “no thirst” and on the right by “intolerable thirst” [14] .

### Neuroradiological examinations

In order to assess post-hypophyseal vasopressin content, brain magnetic resonance imaging (MRI) was performed on a 0.5-T magnet system (MR Max, General Electric, Milwaukee, WI) in patients deemed transportable and having no contraindication. During the MRI, patients were monitored by magnetic system (Maglife ODAM, Bruker, Wissembourg, France). Sagittal and coronal 3-mm thick contiguous sections were obtained by T1-weighted gradient-echo sequences (TR 430 msec, TE 12 msec, flip-angle 90°) of the pituitary gland, with four excitations and a 17-cm field of view, as previously described. In all sagittal imaging, the direction of the frequency encoding gradient was antero-posterior to shift the marrow fat of the dorsum sellae posteriorly. Signal intensity was measured by placing a 1–2 mm^2^ region of interest in the anterior and the posterior lobe, and in the pons [6]. The images were interpreted by one radiologist (R.C.) who was unaware of the patients’ plasma vasopressin levels.

### Data Collection

From admission in ICU, we recorded demographic data, past medical history, estimated prognosis of preexisting underlying illness (graded as nonfatal, ultimately and rapidly fatal disease) [15] , category of ICU admission (medical vs. surgical), the Simplified Acute Physiology Score II (SAPSII) and the sequential organ failure assessment score (SOFA) [16,17] and body weight (Kg). 

From ICU admission to osmotic challenge included, we recorded criteria for septic shock, the primary source of infection, causal micro-organism and positive blood cultures. We recorded for each day vital signs, arterial blood gas, plasma lactate levels, standard laboratory tests, the administration of catecholamines, sedatives and analgesics, the need for any surgical procedure, renal replacement and mechanical ventilation as well as treatment that might alter AVP secretion. Using these data, the maximum SOFA score, lowest PaO_2_/FiO_2_ ratio, duration of shock (i.e. defined as duration of catecholamines infusion), sedation and mechanical ventilation were calculated. 

For the 5 days prior to the osmotic challenge, the maximal and minimal values of blood pressure, body weight, plasma levels of sodium and osmolarity, maximal positive end expiratory pressure (PEEP) and drugs interacting with osmolality or vasopressin secretion (diuretic, anti-psychotics, benzodiazepines, opioids) were also recorded to determine the presence of osmoreceptor and baroreflex mediated factors that could influence the response to osmotic challenge. 

After osmotic challenge, duration of mechanical ventilation, length of stay in ICU and hospital and patients' status at discharge from hospital were recorded.

### Study End Points

The primary end point was to determine the change in plasma AVP levels in relation to an increase in plasma sodium levels during recovery from septic shock. Then, we determined the proportion of patients with normal (responder) and impaired increase in AVP concentrations (nonresponder) and the main clinical and biological differences between them. 

### Statistical Analyses

Our a priori hypothesis was that vasopressin secretion to osmotic challenge was impaired in half of patients recovering from their septic shock. Thus, the number of patients to be included in the present applied physiological study was fixed a priori to 30, based on the following criteria: 1 – Feasability, as the osmotic challenges with repeated assessment of vasopressine cannot be performed in a large cohort of patients; 2 – A sample size of 30 patients would allow an estimate of the prevalence of impaired osmoregulation with a precision less than 20% assuming a similar prevalence as in our previous study of 33 patients tested at the post-acute phase of their septic shock, in which 52% of patients presented an altered vasopressin secretion to osmotic challenge [5]. 

Quantitative variables were expressed as median with interquartile range and qualitative variable as number of patients and percentage. Changes in quantitative variables during osmotic challenge were analysed using mixed models analysis of covariance with random intercept and slopes to account for correlation between data measured on the same subjects. The random effects and residual variance structures were selected, using Schwarz' BIC model selection criterion [18] . The model fit was assessed by examining residuals and whenever useful, variables were log-transformed (this was the case for AVP levels, for instance). Models were fitted using restricted maximum likelihood [19]. However, comparisons of the models involving different fixed effects were based on maximum likelihood estimates, as likelihood comparisons between restricted maximum likelihood fits with different fixed effects are not valid [20]. Specific hypotheses regarding fixed effects were tested, using Wald or likelihood ratio tests. Results are presented as estimates with their 95% confidence interval (CI). 

The distributions of variables were compared between responders and non-responders using nonparametric Wilcoxon rank-sum tests for quantitative variables, and Fisher's exact tests for categorical variables. A p < 0.05 was considered to be significant. Analyses were performed, using R version 2.13.2 statistical package (The R Foundation for Statistical Computing, Vienna, Austria).

## Results

Thirty of 82 screened patients assessed were included, and their characteristics are detailed in Tables 1 and 2. None of our patients received vasopressin infusion. 

**Table 1:** General characteristics of patients.

			**Overall N=30**	**Responders N=12**	**Non-Responders N=18**		**p**
Age (years)			69 (55-85)	84 (69-85)	63 (54-76)		0.08
Women (%)			10 (33.3)	5 (27.8)	5 (41.7)		0.46
McCabe classification (%)							0.44
	0: no fatal underlying disease		19 (63.3)	9 (75)	10 (55.6)		
	1: life expectancy ≤5 years		11 (36.7)	3 (25)	8 (44.4)		
	2: life expectancy <1 year		0	0	0		
Patient's location before ICU admission (%)							0.66
	Home		15 (50)	7 (58.3)	8 (44.4)		
	Ward		15 (50)	5 (41.7)	10 (55.6)		
Pre-existing cardiac insufficiency (%)			6 (20)	3 (25)	3 (16.7)		0.66
Surgical admission (%)			10 (33.3)	4 (33.3)	6 (31.6)		0.99
Weight (kg)			68 (65-79)	67 (61-73)	70 (66-79)		0.29
SAPS II at admission			53 (39-64)	57 (38-80)	52 (39-60)		0.45
SOFA score at admission			10 (6-12)	10 (3-12)	10 (8-12)		0.77
**Characteristics of shock**							
SIRS criteria at admission							
	Temperature (°C)		37.3 (36.2-38.3)	36.5 (36.2-38.0)	37.4 (36.1-38.3)		
	Heart rate (bpm)		108 (90-121)	103 (85-111)	111 (101-124)		
	Leucocyte count (×109/L)		13.9 (10.3-20.1)	14.6 (13.5-16.9)	11.9 (10.2-22.4)		
Tissue hypoperfusion/organ dysfunction							
	PaO2/FiO2 (mm Hg)		189 (121-273)	152 (74-191)	258 (171-300)		0.02
	Urinary output (mL/24 h)		950 (400-1630)	900 (400-1620)	1175(480-1750)		
	Lactate (mmol/L)		2.1 (1.3-4)	2.7 (2.1-3.3)	1.9 (1.1-4.7)		
	Platelet counts (×10^9^/L)		232 (150 -311)	263 (172-325)	200 (130-303)		
	Haematocrit (%)		33.6 (31.3-38)	34.3 (28.8 -39.6)	33.7 (31.4 -37.1)		
	Glasgow Coma Score		9 (3-15)	10 (3-14)	9 (3-15)		
Shock criteria							
	Systolic blood pressure (mm Hg)		90 (84-98)	88 (85-92)	91 (84-99)		
	Mean arterial blood pressure (mm Hg)		63 (59-72)	63 (57-73)	64 (60-68)		
	Catecholamine requirements (%)		30 (100)	12 (100)	18 (100)		
		Epinephrine (%)	7 (23.3)	2 (16.7)	5 (27.8)		
		Norepinephrine (%)	20 (66.7)	8 (66.7)	12 (66.7)		
		Dobutamine and norepinephrine (%)	2 (6.7)	1 (8.3)	1 (5.5)		
		Epinephrine and norepinephrine (%)	1 (3.3)	1 (8.3)	0 (0.0)		
**From onset of shock to inclusion**							
	Duration of shock (days)		4 (3-6)	4 (3-7)	4 (3-5)		
	SOFA max		13 (11-15)	13 (12-16)	12 (10-13)		0.10
	SOFA inclusion		2 (1-4)	2 (1-5)	2 (0-3)		0.31
	Renal replacement therapy (%)		7 (23.3)	4 (33.3)	3 (16.7)		0.39
	Mechanically ventilated patients (%)		28 (93.3)	12 (100)	16 (88.9)		0.7
	Duration of mechanical ventilation		12 (6-18)	16 (10-21)	7 (4-13)		0.12
	P/F min		152 (91-243)	104 (60-168)	211 (105-300)		0.028
	Plasma bilirubin level max (µmol/l)		15 (9-23)	10 (9-14)	21 (12-26)		0.065
	Cumulative doses of catecholamines requirement (mg)		61 (34-170)	114 (35-174)	51 (34-115)		0.4
	Duration of sedation (days)		5 (2-9)	9 (4-19)	4 (2-6)		0.08
	Delay from ICU admission to inclusion (days)		15 (10-23)	18 (11-29)	12 (10-21)		0.31
	Delay from onset of shock to inclusion (days)		12 (10-19)	14 (10-20)	12 (10-18)		0.73
	Delay from end of shock to inclusion (days)		8 (5-11)	8 (6-10)	7 (5-15)		0.47

**Table 2:** Characteristics of infection.

		**Overall N= 30**	**Responders N= 12**	**Non-Responders N= 18**	**p**
**Primary source of infection**					0.52
	Lung (%)	15 (50)	8 (66.7)	7 (38.9)	
	Urinary tract (%)	7 (23.3)	4 (33.3)	3 (16.7)	
	Abdomen (%)	6 (20)	3 (0.25)	3 (16.7)	
	Soft tissues (%)	3 (10)	0	3 (16.7)	
	Catheter related (%)	2 (6.6)	2 (16.6)	0 (0.0)	
	Endocarditis (%)	1 (3.3)	0	1 (5.6)	
**Positive blood culture (%)**		3 (10)	1 (8.3)	2 (11.1)	
**Causal micro organism**					**0.22**
	None (%)	4 (13.3)	2 (16.7)	2 (11.1)	
	One (%)	14 (46.7)	5 (41.7)	9 (50.0)	
	More than one (%)	12 (40)	5 (41.7)	7 (38.9)	
	Gram-negative bacillus (%)	18 (60)	9 (75.0)	9 (50.0)	
	Gram-positive cocci (%)	10 (33.3)	4 (33.3)	6 (33.3)	
	Fungi (%)	2 (6.6)	1 (8.3)	1 (5.6)	
	Anaerobes (%)	2 (6.6)	1 (8.3)	1 (5.6)	

Data are median (IQR) for continuous variables and n (%) for categorical variables

### Whole Population

Osmotic challenge was performed a median 8 days after the resolution of septic shock and was well tolerated by all participants. Median plasma sodium level was 136 mEq/L (interquartile range, 135-138) at baseline and 146 mEq/L (interquartile range, 143-149) at the end of the hypertonic saline infusion. Increase in plasma sodium levels was significant and linear with an average by 4.9 mEq/L per hour (95% CI, 4.4–5.4; p < .0001). In response to the challenge plasma AVP level increased from 2.6 pg/mL (interquartile range, 2.2-3.6) to median 7.1 pg/mL (interquartile range, 4.5-13.9). This increase was not linear in time, and results showed that plasma AVP levels increased 1.6 fold per hour on average (95% CI, 1.4–1.8; p<0.0001). Plasma levels of glucose and urea did not change significantly during osmotic test (p=0.28 and p=0.92). Central venous pressure, measured in the six patients with central venous catheter, remained unchanged during and after the test (median 12.5 vs 13 mmHg).

### Vasopressin Responders Versus Non-responders

Twelve (40%) patients were considered responders and 18 (60%) non-responders. In responders, plasma AVP levels increased on average by 2.2 fold per hour (95% CI, 1.9–2.5), significantly more than in non-responders (on average 1.4-fold increase per hour, 95% CI, 1.2- 1.5, p<0.0001). Baseline plasma vasopressin levels at baseline did not differ between responders and non-responders (mean difference 0.9 pg/ml, 95% CI, -1.3–3.0). 

**Figure 1:**
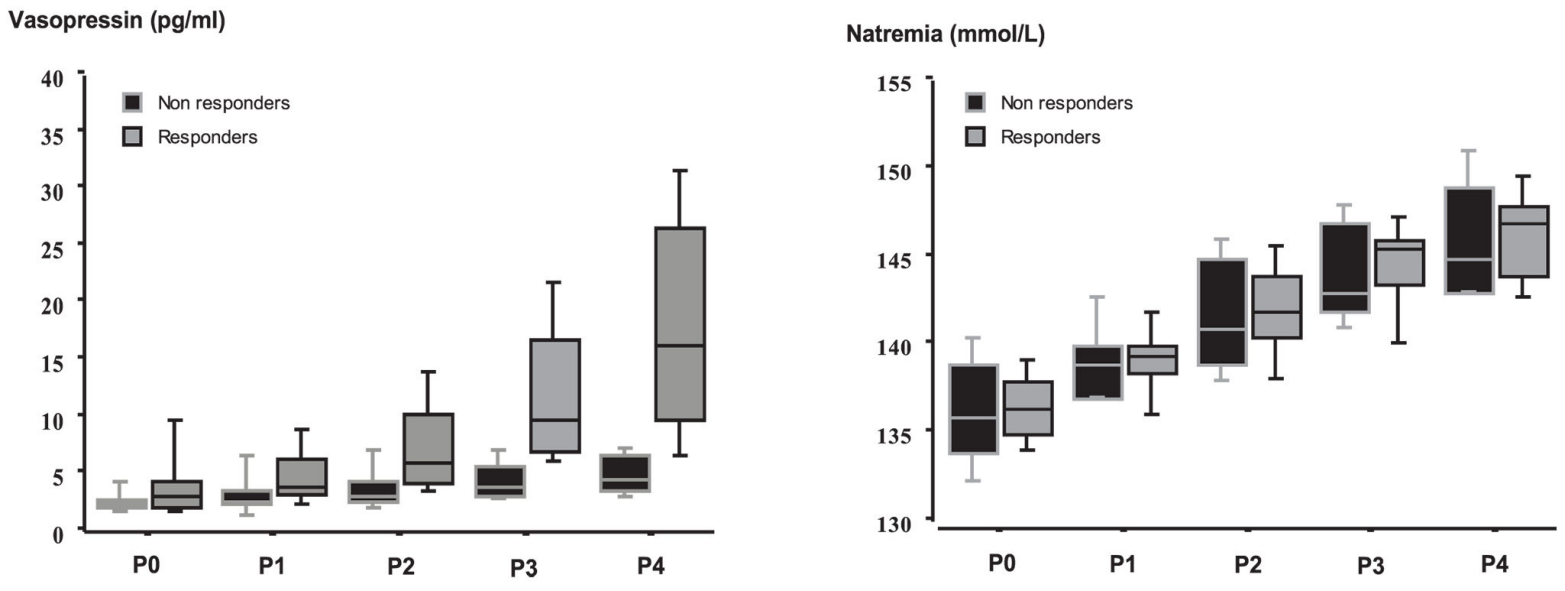
Variations of plasma sodium levels (mmol/L) and plasma vasopressin levels (pg/ml) during the osmotic challenge in responders and non-responders. Measurements were performed 15 mins before (P0) and then 15 (P1), 45 (P2), 75 (P3), and 105 (P4) mins after the beginning of saline hypertonic infusion. During osmotic challenge, changes in plasma sodium levels did not differ between the two groups. Values are expressed as median with interquartile range.

**Figure 2:**
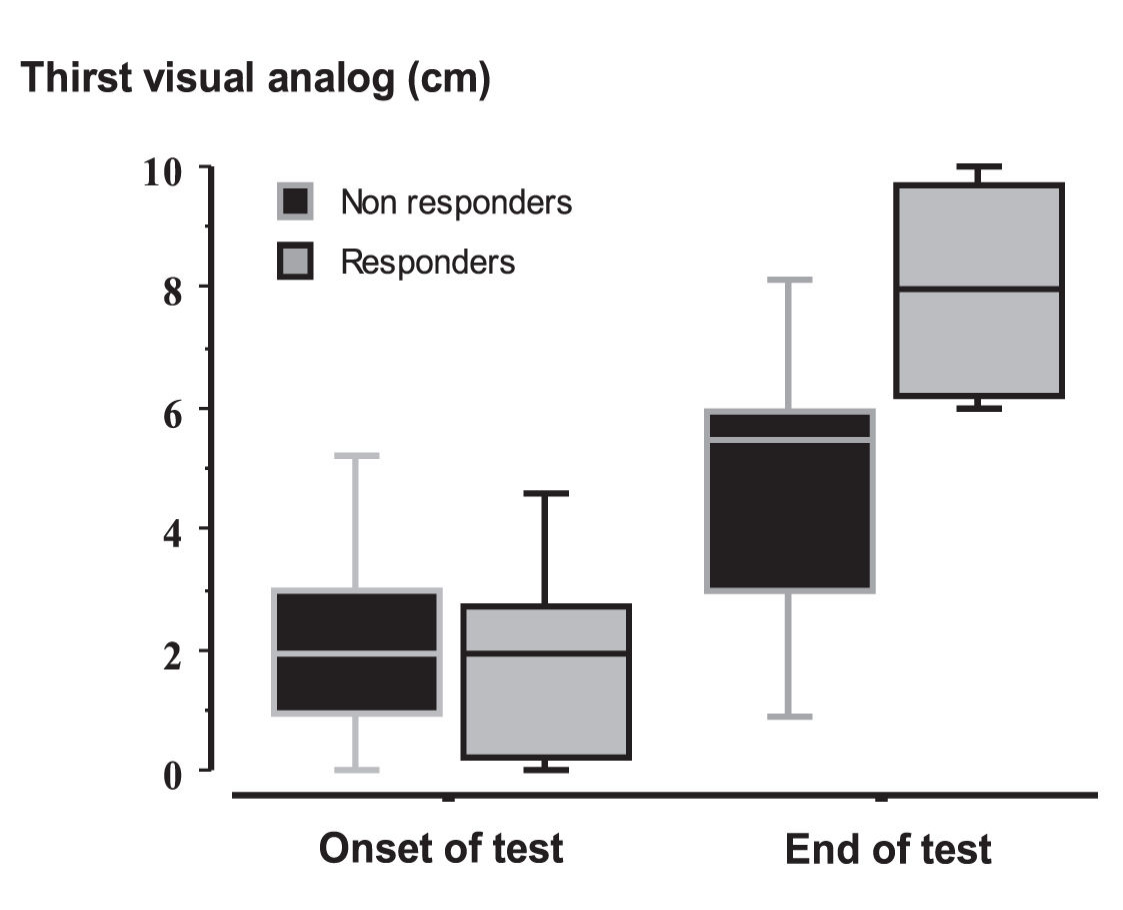
Change in thirst (cm on VAS) during the osmotic challenge in responders and non-responders. Thirst was comparable between the two groups at baseline but was significantly greater in responders at end of test Values are expressed as median with interquartile range.

**Figure 3:**
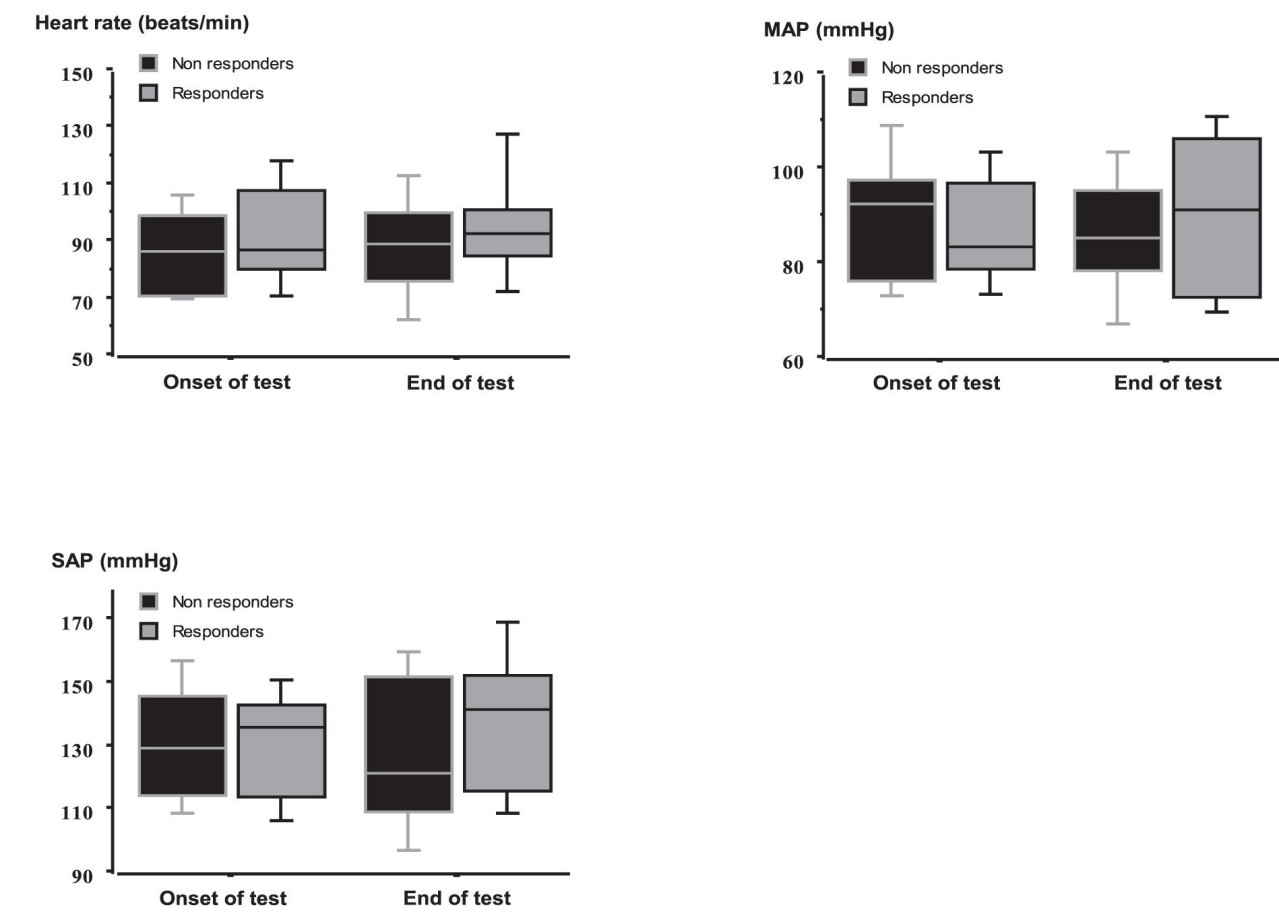
Variations of heart rate (beats/min), mean arterial pressure (MAP) (mmHg) and systolic arterial pressure (SAP) (mmHg) during the osmotic challenge in responders and non-responders. During osmotic challenge, changes in heart rate, SAP, MAP, and plasma levels of BNP did not differ between the two groups Values are expressed as median with interquartile range.

At baseline and during osmotic challenge, plasma sodium levels did not differ between the two groups (Figure 1). VAS thirst was comparable between the two groups at baseline but was significantly greater in responders at end of test (Figure 2 and Table 3). During osmotic challenge, changes in plasma sodium levels, heart rate, SAP, MAP, and plasma levels of BNP did not differ between the two groups (Figure 3 and Table 3). MRI was performed in six patients and showed a decrease in intensity of post-hypophyseal signal, which were comparable between the three responders and the three non-responders. 

**Table 3:** Evolution of parameters during osmotic challenge.

		**Overall N=30**	**Responders N=12**	**Non-Responders N= 18**	**p**
Plasma sodium level (mmol/l)					
	Onset of test	136 (135-138)	137 (135-138)	136 (134-139)	0.97
	End of test	146 (143-149)	147 (145-148)	145 (143-149)	
	Variation of plasma Na	10 (8-12)	10 (9-12)	9 (8-12)	0.67*
Brain natriuretic peptide (ng/ml)					
	Onset of test	380 (178-618)	594 (181-719)	314 (179-553)	0.58
	End of test	336 (170-630)	450 (186-558)	328 (179-514)	0.41
Body temperature (°C)					
	Onset of test	37.1 (36.7-37.5)	37.0 (36.6-37.4)	37.2 (36.8-37.6)	
Plasma creatinine level (µmol/l)					
	Onset of test	69 (51-111)	87 (62-113)	58 (48-103)	
Plasma Cortisol level (µmol/l)					
	Onset of test	155 (144-207)	152 (143-162)	161 (146-217)	
PCT (ng/ml)					
	Onset of test	0.3 (0.1-1.3)	0.3 (0.2-1.9)	0.3 (0.1-0.6)	
Plasma AVP level (pg/ml)					
	Onset of test	2.6 (2.2-3.6)	3.2 (2.3-4.6)	2.5 (2.2-2.9)	0.31
	End of test	7.1(4.5- 13.9)	16.4(10.6-24.6)	4.7 (3.9-6.7)	
	Variation of plasma ADH	3.7 (2.0-9.2)	10.1 (7.4-20.1)	2.1 (1.3-3.0)	<0.0001*
Thirst VAS level (cm)					
	Patients evaluated (%)	21 (70.0)	7 (58.3)	14 (77.8)	
	Onset of test	2.0 (1.0-3.0)	2.0 (0.5-2.5)	2.0 (1.0-2.8)	0.76
	End of test	6.0 (5.0-8.0)	8.0 (6.5-9.5)	5.5 (3.3-6.0)	
	Variation of thirst	4 (1.8-6)	6 (5.5-7.5)	3 (1.3-4)	0.014

Data are median (IQR) for continuous variables and n (%) for categorical variables

* test for equal variation in the mixed models analysis of covariance

Main differences between responders and non-responders are summarized in Tables 1, 2, and 3. In comparison to non-responders, responders had a lower PaO_2_/FiO_2_ ratio and tended to be older, to have been sedated for longer period and to have lower plasma bilirubin levels. Response did not differ between those admitted to ICU from home or from hospital or between medical and surgical cases. SAPS-II at admission did not differ between the two groups as well as, up to inclusion, maximal SOFA score, duration of septic shock, cumulative dose of catecholamines, proportion mechanically ventilated, duration of mechanical ventilation and need for renal replacement. The use of drugs interacting with osmolality or AVP secretion was similar in the two groups (data not shown). 

During the five days before inclusion, variation in body weight, maximal and minimal values of SAP, MAP and plasma levels of sodium and osmolarity were comparable between the two groups as was maximal PEEP value. Change in body weight between admission and inclusion was also not different (Table 4). 

**Table 4:** Parameters known to influence AVP secretion within the 5 days before inclusion.

		**Overall N=30**	**Responders N=12**	**Non-Responders N= 18**	**p**
Variation of weight (kg)		1.8 (0.3-3.4)	1.1 (0.1-3.8)	2 (0.9-3.2)	
Mechanically ventilated patients (%)		21 (70.0)	9 (75.0)	12 (66.7)	
PEEP max (mmHg)		7 (5-8)	7 (5-7)	7 (5-8)	
Mean urinary output (ml/day)		2000 (1430-2700)	2140 (1400-2890)	1960 (1430-2450)	
Na (mmol/l)					
	Max	141 (140-145)	142 (140-143)	141 (140-147)	0.73
	Min	137 (135-139)	137 (135-137)	136 (135-139)	0.81
Osmolarity (mosmol/l)					
	Max	306 (299-319)	306 (303-324)	308 (299-316)	
	Min	295 (286-307)	297 (289-307)	295 (285-305)	
SAP (mmHg)					
	Max	164 (150-186)	170 (159-184)	160 (147-186)	
	Min	102 (90-113)	105 (95-110)	101 (87-114)	
MAP (mmHg)					
	Max	114 (105-131)	114 (109-126)	116 (99-131)	
	Min	69 (60-76)	71 (63-75)	66 (60-78)	
Sedation requirement (%)		7 (23.3)	3 (25.0)	4 (22.2)	
	Midazolam (%)	6 (20)	3 (25.0)	3 (16.7)	
	Sufentanyl (%)	7 (23.3)	2 (16.7)	4 (22.2)	
Use of diuretics		13 (43.3)	7 (58.3)	6 (33.3)	

### Outcome and repeated assessment of osmoregulation

Eight (27%) patients died in the ICU (Table 5). In-hospital mortality rate did not differ between the two groups as well as lengths of stay in the ICU and in hospital. 

**Table 5:** Outcome of patients after osmotic challenge.

	**Overall N= 30**	**Responders N= 12**	**Non-Responders N= 18**	**p**
**Hospital mortality (%)**	8 (26.7)	5 (41.7)	3 (25.0)	0.2
**Nosocomial infections (%)**	12 (40)	5 (41.7)	7 (38.9)	
**Length of stay in the ICU after test (days)**	3 (1-20)	5 (2-20)	3 (0-15)	
**Length of hospital stay after test (days)**	12 (6-21)	12 (3-21)	13 (7-22)	

Osmotic challenge was performed in five patients (1 responder and 4 non responders) a median 6 months after hospital discharge. There was no change in the category of response between the first and second tests. 

## Discussion

The first finding of the present study is that AVP secretion in response to an osmotic challenge is dramatically impaired in 60 percent of patients recovering from septic shock (i.e. 5 days from catecholamines discontinuation). Also, although assessed in a limited number of patients this pattern appears to persist long after hospital discharge. In our previous study, AVP secretion did not increase in half of patients *osmotically* challenged in the post-acute phase of their septic shock (i.e. 3 days after onset) [5]. Taken together, these results indicate that this is an early and protracted phenomenon. The other findings are that perception of thirst was decreased in non-responders and that post-hypophyseal AVP content on MRI was depleted in both responders and non-responders, providing new insight into the mechanisms of this neuroendocrine dysfunction. 

The decrease in thirst perception suggests that osmoreceptors are functionally or structurally altered. Indeed, pathways to thirst centre and to hypothalamic paraventricular and supra-optic nuclei are originating from osmoreceptors, which are specialised neurones of the circumventricular organs sensitive to osmotic variation, *via* membrane depolarisation secondary to activation of a non-selective cation current [7]. The mechanisms of osmoreceptors dysfunction can be only speculative and their investigation would require *in-vitro* or *in vivo* studies. However, one may argue that the persistence of alteration of osmoregulation months after septic shock suggests that pool of osmoreceptors has been diminished, notably secondarily to apoptosis that has been evidenced in hypothalamic neurones during septic shock [21]. Additionally, there are many biological factors that can modulate osmoreceptors activity [22,23], notably factors that are specifically related to pneumonia and liver failure. 

Indeed, we confirmed our previous findings that non-responders tended to have had more severe liver failure (as suggested by the increase in plasma bilirubin levels) while responders had more severe lung injury. Alteration of osmoregulation in liver failure has not been reported, to our knowledge. Interestingly, osmoreceptors are also expressed in the hepatic portal vein [24,25] and it is plausible that a common mechanism affects both hepatic and brain osmoreceptors. Pneumonia is a known cause of inappropriate antidiuresis syndrome (SIAD) [26,27], which may have facilitated both baseline and osmoreceptor-mediated AVP secretion. The mechanisms of pneumonia associated SIAD are unknown, and may involve baroreflex, hypoxemia or inflammatory mediators, although none of these factors differed between responders and non-responders. Indeed, PaO_2_ values were comparable as well as changes in blood pressure, heart rate and plasma levels of BNP. This makes it unlikely that the baroreflex pathway had been less stimulated in non-responders, or at least in range that could blunt response to osmotic challenge. It has to be noted that increase in circulating volume or blood pressure shifts rather than inhibiting the response [28]. Moreover, the duration and severity of septic shock were not statistically different, suggesting that the inflammatory insult would have been comparable between responders and non-responders.

Although performed in few patients, MRI indicates that the pattern of response to osmotic challenge did not depend on post-hypohyseal AVP content, which was depleted in both responders and non-responders. The most plausible explanation is that hypotension, at onset of septic shock, had durably depleted the posterior-hypophysis in all patients. This neuroradiological finding does not therefore contradict our hypothesis of osmoreceptor dysfunction in non-responders. 

We found that alteration of osmoregulation of AVP secretion, which is a major homeostatic function, was not associated with an increased mortality or morbidity. The study was not powered or designed to address this issue. It is plausible that non-responders might be at higher risk of death. It has been recently shown that hypernatremia was associated with increased mortality in critically ill patients [29]. Hypernatremia is probably multifactorial and might be partly related to osmoregulation dysfunction. On the other hand, non-responders might have developed compensatory mechanisms that allow them to survive, as suggested by the fact that osmoregulation impairment can persist months afterward. It would be of interest to investigate this compensatory mechanism, which notably might involve renin-angiotensin system [30]. In addition, vasopressin is a neurotransmitter, which plays an important role in control of cognition and behaviour, especially libido and emotion [31,32]. One may argue that impairment of osmoregulation of vasopressin secretion is one part of a more diffuse vasopressin deficiency within the brain. However, we are not able to address this hypothesis as we have not assessed psychological status or cognitive function in our patients. 

### Methodological issues

There were some discrepancies between our previous [5] and present studies. Thus in non-responders, plasma AVP levels at baseline were previously lower and the rate of ICU admission from ward and delay between ICU admission and inclusion were greater. The difference in baseline plasma AVP levels is likely related to the fact that our two studies were performed at two separate times of septic shock course (i.e. post-acute and recovery phases) during which plasma vasopressin level has been shown to decrease [4]. The difference in admission and time to inclusion in our previous study had been considered an argument that non-responders had a longer duration of sickness and critical illness. In the present study, this temporal confounder is ruled out as osmotic test has been performed at the same time of hospital and ICU stay as well as sepsis course in all patients, responders or not. 

The osmotic challenge was well tolerated and achieved and in all cases increased plasma sodium level within the range that has been demonstrated to stimulate AVP release in healthy subjects [33]. More importantly, variation in natremia was comparable between responders and non-responders, indicating that osmoregulation-mediated AVP secretion had been equally solicited. Variation in natremia was the only determinant of change in osmolality as both plasma levels of glucose and urea did not change during the osmotic challenge. Once again, the absence of difference in blood pressure, heart rate and plasma levels of BNP indicates that baroreflex pathway had been equivalently stimulated in responders and non-responders. 

In order to be consistent and allow comparison with our previous findings, we have used the same definition of normal response. This cut-off was derived from data obtained, with help of an equivalent osmotic test, in both and young healthy subjects [13]. These physiological studies reported that a slope of plasma AVP was always above 0.5. These results indicate that our cut-off was not arbitrary and the osmotic test has been well-validated, making not necessary to assess osmotic response in a new set of healthy subjects. It must be emphasized that the osmotic challenge used in this study was comparable to that performed in patients with insipidus diabetes or SIAD [9,12,13]. We acknowledge that this criterion (i.e. slope > 0.5) has been established in healthy subjects. Indeed, the translation of any criteria of normality established in homeostatic (physiological) condition into an allostatic (pathophysiological) one is open to challenge. By using this definition, there were no in-between subjects (or ambiguous response) as responders and non-responders dose-response curves were clearly separated. It enables to determine predisposing factors such as pneumonia for preserved response and liver failure for altered response [5]. On the other hand, one may argue that this dichotomy did not identify a major and relevant disturbance as response pattern did not appear to alter risk of death or complication. Our study was not powered to address this outcome but rather designed for assessing the physiological response. 

One may argue that we should have assessed osmotic response in non-septic critically ill patients, in order to determine if the disorder of osmoregulation is specifically related to sepsis. We think that this would be an interesting future and complementary study but that it is not useful at this stage of our research project. Our work has been focused on septic shock patients because it is in this group that so-called “AVP relative deficiency” was originally reported [1]. This finding led us to assess osmoregulation of AVP secretion firstly during post-acute phase of septic shock [5] and herein during recovery phase. 

One may argue that impaired osmotic response might have resulted from the persistence of endotoxemia. This is highly unlikely, although we are not able to exclude this possibility as presence of circulating endotoxin has not been assessed. Indeed, the absence of clinical signs of infection and the low plasma level of Procalcitonin at time of test indicates that sepsis was satisfactorily controlled. 

Finally, it seems very unlikely that impairment of osmoregulation preceded the onset of septic shock. Indeed, an incidence of osmoregulation impairment over 50 percent has never been reported in the general population [33]. Moreover, in the present and previous studies [5], patients with neuroendocrine or neurological disease were excluded and non-responders tended to be younger and were not more frequently treated with drugs interfering with AVP secretion. 

In conclusion, we found alteration of osmoregulation of AVP release, characterized by a dramatic decrease in vasopressin secretion and thirst perception during osmotic challenge, in almost two-third of patients recovering from septic shock. Non-responders had no specific features whereas gas exchange was more severely impaired in responders. Osmoregulation can be altered months after ICU discharge. These findings raise several pathophysiological issues: in responders, on a potential pulmonary factor; in non-responders, on factors altering osmoreceptors but also on compensatory mechanisms that would account for patients surviving with such neuroendocrine dysfunction. 
